# Computational Structure-Based *De Novo* Design of Hypothetical Inhibitors against the Anti- Inflammatory Target COX-2

**DOI:** 10.1371/journal.pone.0134691

**Published:** 2015-08-04

**Authors:** Jaspreet Kaur Dhanjal, Anith Kumar Sreenidhi, Khushboo Bafna, Shashank Prakash Katiyar, Sukriti Goyal, Abhinav Grover, Durai Sundar

**Affiliations:** 1 Department of Biochemical Engineering and Biotechnology, Indian institute of Technology Delhi, New Delhi, India; 2 Department of Biotechnology, School of Life Sciences, University of Hyderabad, Hyderabad, India; 3 Apaji Institute of Mathematics & Applied Computer Technology, Banasthali University, Tonk, Rajasthan, India; 4 School of Biotechnology, Jawaharlal Nehru University, New Delhi, India; Wake Forest University, UNITED STATES

## Abstract

Cyclooxygenase-2 (COX-2) produces prostaglandins in inflamed tissues and hence has been considered as an important target for the development of anti-inflammatory drugs since long. Administration of traditional non-steroidal anti-inflammatory drugs (NSAIDs) and other COX-2 selective inhibitors (COXIBS) for the treat of inflammation has been found to be associated with side effects, which mainly includes gastro-intestinal (GI) toxicity. The present study involves developing a virtual library of novel molecules with high druglikeliness using structure-based *de novo* drug designing and 2D fingerprinting approach. A library of 2657 drug like molecules was generated. 2D fingerprinting based screening of the designed library gave a unique set of compounds. Molecular docking approach was then used to identify two compounds highly specific for COX-2 isoform. Molecular dynamics simulations of protein-ligand complexes revealed that the candidate ligands were dynamically stable within the cyclooxygenase binding site of COX-2. The ligands were further analyzed for their druglikeliness, ADMET properties and synthetic accessibility using knowledge based set of rules. The results revealed that the molecules are predicted to selectively bind to COX-2 enzyme thereby potentially overcoming the limitations posed by the drugs in clinical use.

## Introduction

Cyclooxygenase (COX) is an enzyme responsible for catalyzing the conversion of arachidonic acid (AA) to prostaglandin G2 (PGG2) and prostaglandin H2 (PGH2). COX enzymes have two different active sites, each of which performs an important step in the synthesis of prostaglandins. The cyclooxygenase activity of COX firstly oxygenates AA to PGG2 [1–3]. This reaction is catalyzed by activated tyrosly radical of the enzyme (Tyr 371 in COX-2) that converts AA to arachidonyl radical [4]. This arachidonyl radical then reacts with two molecules of oxygen to produce PGG2. The peroxidase activity further reduces PGG2 to PGH2, the root prostaglandin from which other prostaglandins are derived. These prostanoids mediate numerous physiological and pathophysiological effects such as pain, fever, inflammation, homeostasis, regulation of renal function, maintenance of mucosal integrity in the stomach, blood clotting, ovulation, initiation of labor, bone metabolism, nerve growth and development and wound healing [3, 5].

There exist two different isoforms of COX enzyme: COX-1 and COX-2. COX-1 and COX-2 have high degree of structural homology with amino acid identity of 60% [1, 3]. The residues that form the substrate binding catalytic cleft and the channel leading to this site are conserved in both the enzymes except for the substitution of valine with isoleucien in the binding cavity of COX-2. This substitution at position 509 (in human COX-2) results in deletion of methylene group giving rise to a larger binding site in COX-2. This increase in size of the cavity allows the ligands to access the additional pocket that leads directly to solvent [6]. Although quite similar in structure, they significantly differ in their expression profiles. COX-1 is ubiquitous and primarily involved in maintaining the house keeping functions. The major function of COX-1 is to provide PG precursors for homeostatic regulation. On the other hand, COX-2 is induced by stimuli related to inflammatory responses. Increased expression of COX-2 is responsible for an upsurge in prostaglandin production in inflamed joint tissues inducing pain. Pain receptors are sensitive to very low levels of prostaglandins produced in the presence of COX-2. Overabundance of prostaglandins produced by COX-2 relays an improper cellular signal, stimulating improper cell growth and reducing the cleansing effect of apoptosis [7]. Thus, inhibition of COX-2 can effectively lead to anti-inflammatory effects [2, 8].

The origin of non-steroidal anti-inflammatory drugs (NSAIDs) to treat inflammation dates back to 1899 when aspirin was introduced in the market by Bayer. For several centuries NSAIDs have constituted an important class of drugs for inhibiting the enzyme COX [9]. But occurrence of severe gastrointestinal toxicity in patients being treated with these NSAIDs limited their widespread therapeutic use. These toxic effects were attributed to simultaneous inhibition of COX-1 isozyme while attempting to block the functional activity of COX-2 [9, 10]. This raised the need for the development of COX-2 selective inhibitors, COXIBs with enhanced anti-inflammatory and analgesic properties, so as to overcome the limitations associated with the use of NSAIDs. Bextra (valdecoxib), Celebrex (celecoxib), and Vioxx (rofecoxib) are the three COXIBs which were approved by the Food and Drug Administration for clinical use. However, Bextra and Vioxx were later withdrawn from the market because of associated adverse side effects. These side effects were related to gastrointestinal problems and cardiovascular events. Therefore, inspite of tremendous progress made in developing anti-inflammatory agents, design of safe and economical drugs for treating inflammation is still a major challenge. Hence, there is an urgent need to develop novel anti-inflammatory drugs highly specific towards COX-2.

In the present study, we have used *de novo* structure based drug designing method to build a virtual library of ligand molecules having altogether novel chemical scaffolds. These molecules were computationally built within the constraints of binding pocket of COX-2 enzyme by assembling various small structural fragments obtained using existing drugs. Then using molecular modelling techniques, we have reported two COXIBs from the ligand library which were drug-like and highly selective for COX-2 enzyme. These two drug candidates were also compared with the above mentioned FDA approved COXIBs.

## Computational Methods

### Preparation of the human COX receptors

Since the tertiary structure of human COX-1 and COX-2 protein was not available in Protein Data Bank, structures were generated using homology modelling technique. The amino acid sequence of the two proteins was retrieved from UniProt database (UniProt ID: P23219 and P35354). These sequences were searched against the Protein Data Bank entries using protein-protein BLAST algorithm to identify a set of proteins with known crystal structure having close homology to our target sequences [11]. Templates were chosen from the hits obtained and ten comparative models for each protein were generated using the program MODELLER v9.11. The heme group and the ligands were overlaid in their corresponding positions [12]. The quality of the modelled structures was assessed based on the MODELLER DOPE score and molpdf score. Structures were further evaluated using Verify3D score, ERRAT score and Ramachandran maps from the SAVS server [13, 14]. The best scored structure was selected and further refined using simulated annealing and energy minimization process in the Swiss PDB Viewer [15].

### Stabilization of the modeled structures using molecular dynamics simulations

Desmond molecular dynamics software package was employed for all molecular dynamics simulations (MDS) as in our previous study [16–20]. The dynamic behavior of the structures was studied in water-based system. Optimized Potentials for Liquid type Simulations (OPLS)-All Atom force field 2005 was used to perform all MDS steps [21–23]. The modeled structure of the protein was prepared for MDS using the Schrödinger’s Protein preparation wizard to fix the irregularities in the protein structure at atomic level by removing all steric clashes, adding and optimizing hydrogen atoms, checking the bond orders, etc. A cubic periodic boundary box was created around the prepared structure and solvated with TIP3P water model [24]. The solvated system was then neutralized by addition of appropriate number of counter ions. The system was further minimized using steepest descent method for a maximum of 5000 steps or until a gradient threshold of 25 Kcal/mol/Å was reached. It was followed by L-BFGS (Low-memory Broyden-Fletcher-Goldfrab-Shanno quasi-Newtonian minimizer) until a convergence threshold of 1 kcal/mol/Å was reached. Finally, the system was equilibrated using default parameters and simulated with a time step of 2fs. The system was maintained at constant temperature and pressure of 300 K and 1 atm respectively. Smooth Particle Mesh Ewald method was used to calculate long-range electrostatic interactions. 9 Å cut-off radius was used for columbic short-range interaction cut-off method. The structures were simulated for around 15ns.

Root Mean Square Deviation (RMSD), Radius of Gyration (ROG) and Root mean square fluctuation (RMSF) of all the residues of the modeled protein was calculated for the modeled protein to check for its stability and compactness during the simulation. From the stable region of molecular dynamics trajectory, a representative structure was obtained by averaging the coordinates of stable trajectory to be used for further computational studies.

### Designing of compound library

**Fig 1:**
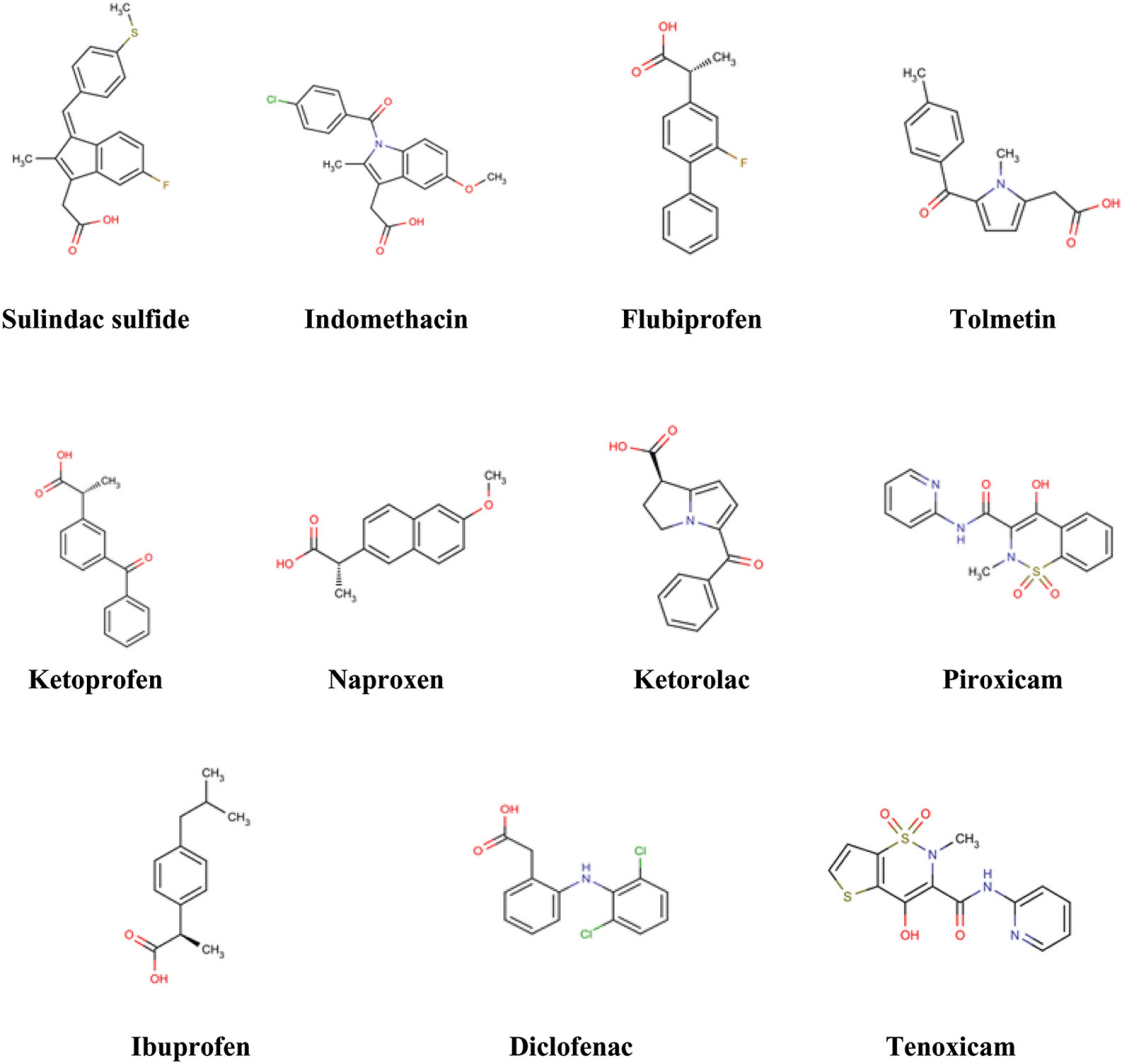
Eleven drug molecules developed against COX-2 which were used for the generation of seed structures in LigBuilder.

LigBuilder program was used to generate a library of molecules from a set of organic fragments [25]. Eleven potent and moderately selective COX-2 inhibitors (Fig 1) were selected for the development of COX-2 inhibitory seed structures. The 3D chemical structures for these drugs were retrieved from NCBI–PubChem and minimized in Tripos Sybyl under Tripos force field [26]. From these eleven drugs, seed structures were generated using “extract” program in LigBuilder. These seed structures were then passed on to the “build” program of LigBuilder, where growing and linking modules were used to generate a large set of molecules using genetic algorithm (GA) embedded within LigBuilder. Chemical rules were simultaneously used for evaluating the "drug-likeness" of the resultant molecules [27, 28]. Based on the evaluated properties, some of the molecules from each of the module were recommended by LigBuilder.

These derivatives were then analyzed to find out similarities within them. This was done by implementing a 2D fingerprinting index *i*.*e*., by calculating Tanimoto coefficients *[29, 30]*. Based on the method described by Peter Willett, an algorithm was developed for calculating Tanimoto coefficients from SMILES format of these structures [30, 31]. Open Babel software was used to obtain SMILES of the molecules generated from LigBuilder [32].

### Identification of COX-2 selective inhibitors using molecular docking

The recommended molecules of the newly designed library were prepared for docking study using LigPrep utility of Schrodinger’s package [33]. It generates all possible tautomeric, stereochemical and ionization variants of the input molecules followed by energy minimization to obtain structures with optimized geometry. The atomistic irregularities in the modelled COX-1 and COX-2 structure were corrected using Schrodinger’s protein preparation wizard [34, 35]. It involves addition and optimization of hydrogen bonds, removal of bad contacts, optimization of bond lengths, creation of disulfide bonds, capping of protein terminals, conversion of selenomethionine to methionine and fixing of missing residues. The prepared structures were then optimized to acquire an energetically stable conformation.

The prepared small molecules were docked against the stabilized human COX-2 structure using Glide software of Schrodinger package [36–39]. A grid was generated around the cyclooxygenase active site of the protein. Docking was carried out using the extra precision (XP) docking protocol of Glide. To find out COX-2 selective inhibitors, the top scoring hits were again docked against the cyclooxygenase cleft of human COX-1 enzyme. The molecules which showed least affinity or had less docking score were then chosen for further study.

The residues of COX proteins involved in interaction with the identified inhibitors were studied using Ligplus program [40].

### To study the dynamic stability of the docked compounds

Protein-ligand complexes were again simulated for 50 ns each in water box using the protocol discussed above to study their dynamic behaviour. The simulated structures were then used to study the changes in the molecular interactions between the ligand and the protein using Ligplus.

### Calculation of binding free energy of the predicted ligands using MM-GBSA method

MM–GBSA method of Schrodinger software which uses a single minimized protein–ligand structure instead of various frames derived from MD trajectories is an efficient method to refine and rescore docking results and calculate free binding energies [41–44]. ΔG_binding_ was calculated for the protein-ligand complexes using MM-GBSA analysis available in prime module of Glide [45, 46]. ΔG_binding_ is calculated based on following formula:

ΔG_binding_ = Energy of the minimized complex–(Energy of the minimized ligand + Energy of the minimized receptor)

### Comparison of the proposed COX-2 inhibitors with already documented drugs

Although great work is being done to develop highly specific COX-2 inhibitors, only three drugs, Bextra (valdecoxib), Celebrex (celecoxib), and Vioxx (rofecoxib) were able to reach market after FDA approval. The identified COXIBs were compared to these drugs in terms of their docking score to predict their action potential. The three dimensional structure of valdecoxib, celecoxib and rofecoxib was retrieved from PubChem and prepared using LigPrep. After preparation the structures were docked against the active site of COX-2 using the same protocol as of identified COXIBs.

### Prediction of druglikeness and ADMET properties of the two ligands

The three dimensional structure of the two predicted ligands were analysed to assess their various physico-chemical properties responsible for making them potential drug like candidates. Their absorption, distribution, metabolism, excretion and toxicity (ADMET) properties were calculated *in silico* using Qikprop module [47] of Schrodinger and an online server called admetSAR [48].

### Prediction of synthetic accessibility of the predicted compounds

Since the reported COX-2 inhibitors were designed using *de novo* approach, the feasibility of their chemical synthesis is undetermined. To avoid the failure of compounds at later stages, due to difficulty in their synthesis, the ease of their synthesizability was predicted using ‘SyntheticAccessibility’ module of myPresto (Medicinally Yielding PRotein Engineering SimulaTOr) program suite [33]. The SA (Synthetic Accessibility) score of all the compounds, including the drugs molecules used for building the compound library, FDA approved drugs against COX and the newly predicted compounds was calculated and ranked between 1 (easy to synthesize) and 10 (difficult to synthesize).

## Results and Discussion

### Preparation of modelled structure of COX-1 and COX-2

Proteins with known crystal structure were selected as template for homology modeling of COX enzymes based on the BLAST search against the PDB database. PDB structures, 1CQE and 1PXX were selected as templates for COX-1 and COX-2 respectively. 1CQE showed 93% sequence similarity with COX-1 with query coverage of 96% while 1PXX had a sequence similarity of 87% with COX-2 covering the complete sequence. Most residues were observed to be conserved in the template and target sequence alignment. Three dimensional structures were then generated using simple template modeling approach of modeller program. Ten structures for each isoform were generated and compared based on least DOPE score to choose final structure of each. The final structures were further checked for their quality. Φ and Ψ angles of all residues of the modeled structures were lying in the allowed regions of the Ramachandran plot. The models were also evaluated using Verify 3D and Errat program. More than 85% of the residues had an averaged 3D-1D score > 0.2. The overall quality factor of both the models was above 80. Verify 3D and Errat scores thus suggested that the quality of the models were comparable to refined structures.

**Fig 2:**
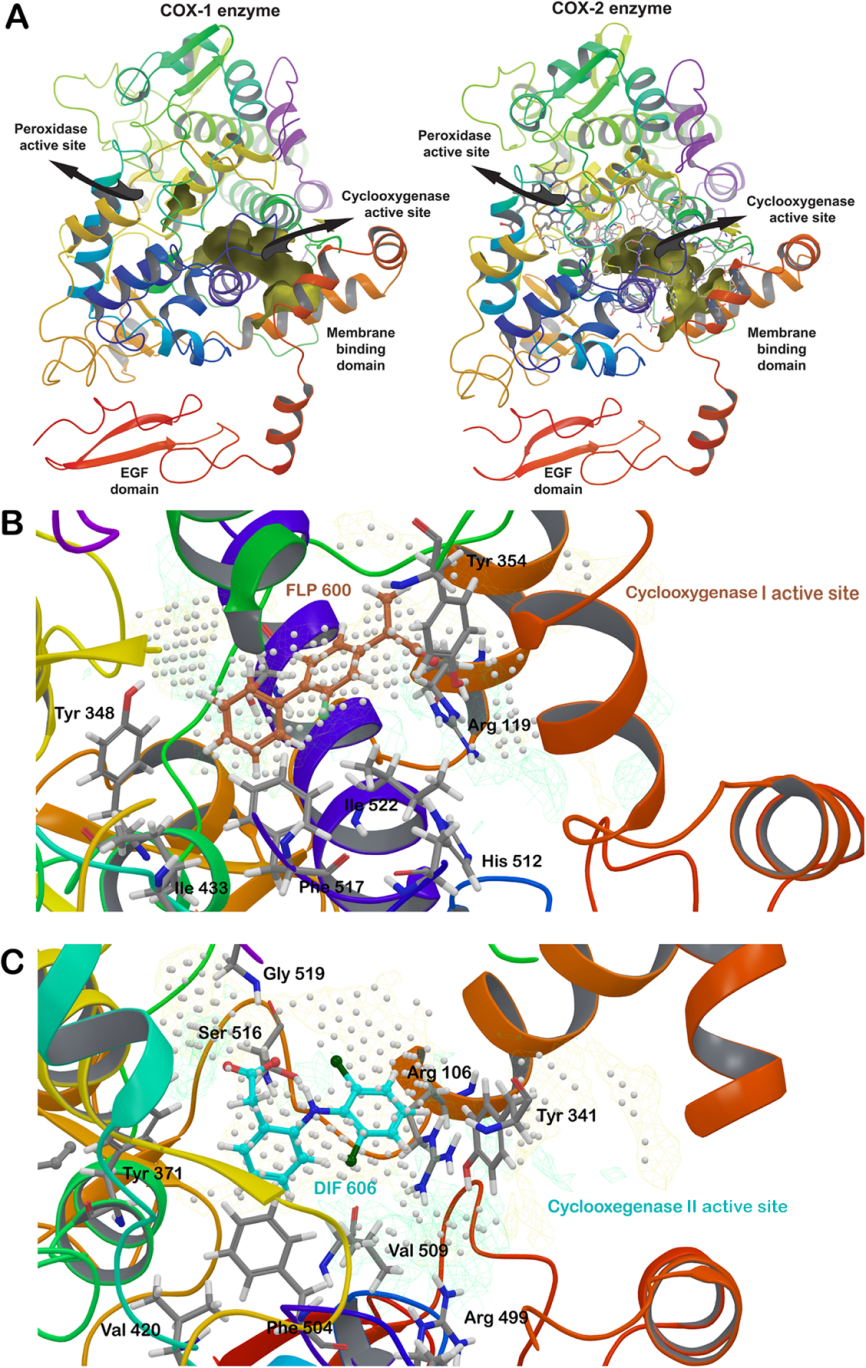
Modelled structure of COX-1 and COX-2. **Comparison of modelled structures with their template structures revealed the active sites of located in the enzymes.** (A) Membrane binding domain, epidermal growth factor binding domain, peroxidase active site, and cyclooxygenase active site of modeled COX-1 and COX-2 enzymes. Shape of cyclooxygenase active sites are shown in yellow and four helices of membrane binding domains are located just beneath it, represented in red. A heme group was present at the peroxidase active site of COX-2 enzyme. (B) Flurbiprofen (brown) was inherited at the cyclooxygenase active site of COX-1 modelled structure from its template structure (PDB ID: 1CQE). Area of active site is depicted by the use of white dots, yellow mesh surface shows hydrophobic region and green mesh surface shows hydrophilic region of the active site. (C) Diclofenac (cyan) was inherited at the cyclooxygenase active site of COX-2 modelled structure from its template structure (PDB ID: 1PXX). Same colours were used to show the area of active site, hydrophobic region and hydrophilic region of the active site. Green hydrophilic channel in COX-2 was larger than the channel of COX-1 enzyme.

Modelled structure of COX-1 and COX-2 was compared with the structure of its template protein to identify and verify various structural details. Both the enzymes had two adjacent active sites within the catalytic domain: peroxidase active site and cyclooxygenase active site. Over all, an epidermal growth factor binding domain, a membrane binding domain and two catalytic domains were identified in the modelled structure of COX-1 and COX-2 (Fig 2(A)). Key amino acids constituting the active site of human COX-1 cyclooxygenase active site include Arg119, Tyr354, Tyr384, Ile433, His512, Phe517 and Ile522 while respective active site amino acids in human COX-2 are Arg106, Tyr341, Tyr371, Val420, Arg499, Ser516, and Gly519 (Fig 2(B) and 2(C)).

### Protein stabilization dynamics

**Fig 3:**
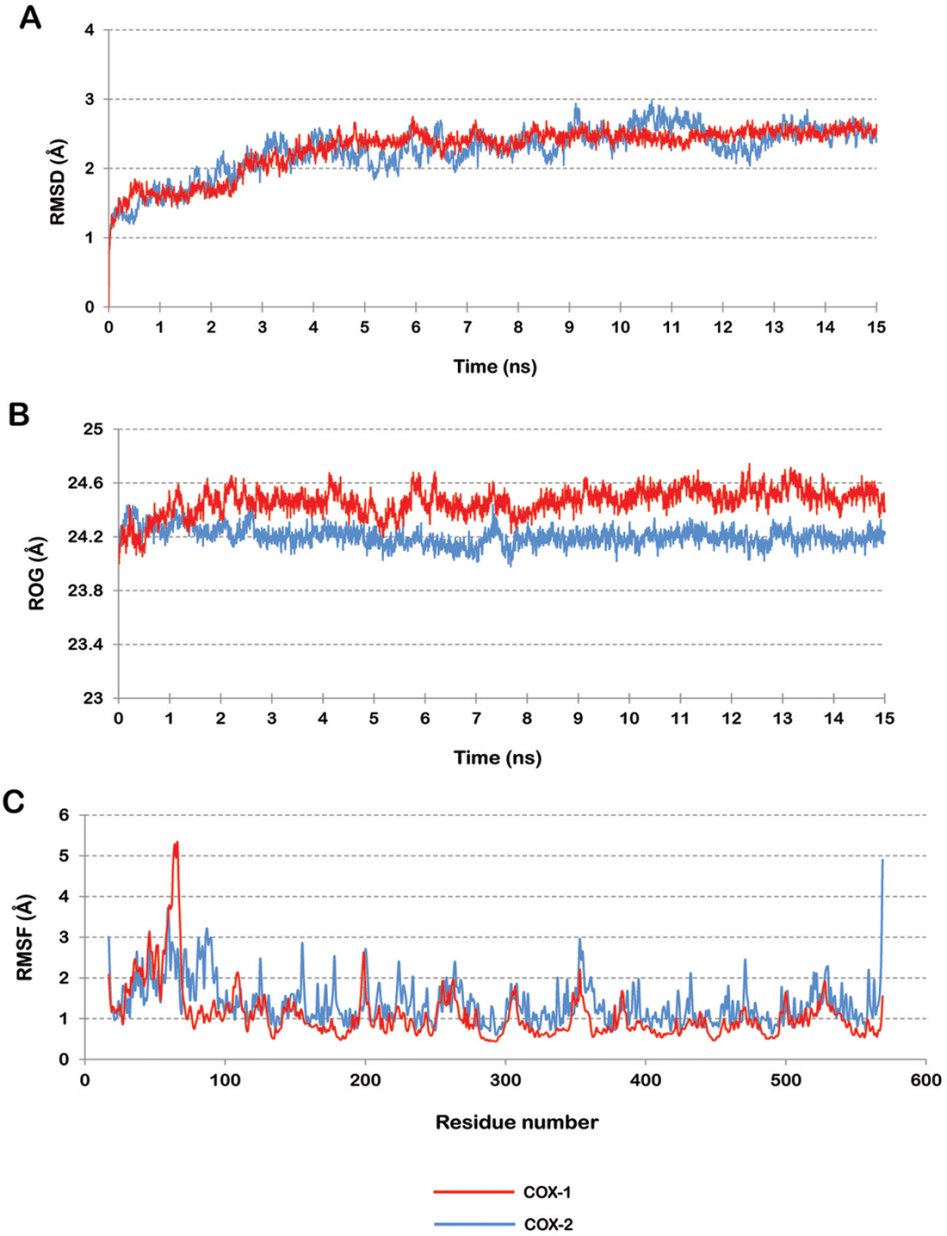
(A) RMSD of the protein backbone in reference to the first frame for COX-1 and COX-2. (B) Radius of gyration for COX-1 and COX-2 over the entire simulation trajectory. (C) RMSF plot for COX-1 and COX-2 protein.

The modelled structures were simulated using water solvent model. A stable trajectory was observed after the completion of 15ns MDS for both the modelled proteins. Backbone of both the proteins, COX-1 and COX-2 showed deviation up to 2.5 Å as shown in Fig 3(A). But it acquired a stable conformation after 6ns of MD run in both the cases. The modelled proteins had a fluctuating ROG of 24.2 Å at the beginning of the simulation (Fig 3(B)). At the end of 15ns simulation the ROG had slightly increased to 24.5 Å in COX-1 protein model and remained approximately the same as the initial structure in case of COX-2 protein model. Hence the ROG analysis suggests that there has been very little change in the compactness of the protein structures during the simulation indicating strong structural stability of the modelled proteins. The RMSF of all the protein residues in both the models showed that the first 100 residues along the terminal regions were quite flexible, while other residues did not show much fluctuation contributing to the stability of the protein structure (Fig 3(C)). The fluctuations in this region were not affecting the conformation of the active cleft. The above observations with consistent RMSD and ROG during the last 8ns indicate that the modelled structures had attained a stable conformation by the end of 15ns MDS.

### Library of molecules

37 seed structures generated from the 11 considered drugs were passed to the “build” program of LigBuilder. In the growing module, the designing of ligand molecules starts with placement of a seed structure with the active pocket of the protein. The program then progressively replaces the other assigned growing points to complete the structure of the molecules. The cycle continues to give a large number of compounds. The 3D coordinates of a total of 1522 molecules were generated using this growing strategy. In the linking process, various seeds are placed at the active points in the catalytic site in a way so as to maximize the interaction between the protein and the ligand in parts. Each seed is then grown and linked to the other one making rational bonds. 1135 virtual molecules were produced in total by this linking mode. Then using the Tanimoto coefficients’ algorithm, it was found that all the molecules were dissimilar with a probability percentage of less than 0.77 and the least similarity was found to be 0.12. This 2D fingerprinting strategy ensured that the library designed was a set of unique 2657 drug-like molecules. Based on calculated physio-chemical properties and estimated binding affinity with the protein, LigBuilder recommended some top scoring compounds as probable inhibitors of COX enzyme.

### Molecular docking and interaction analysis

**Fig 4:**
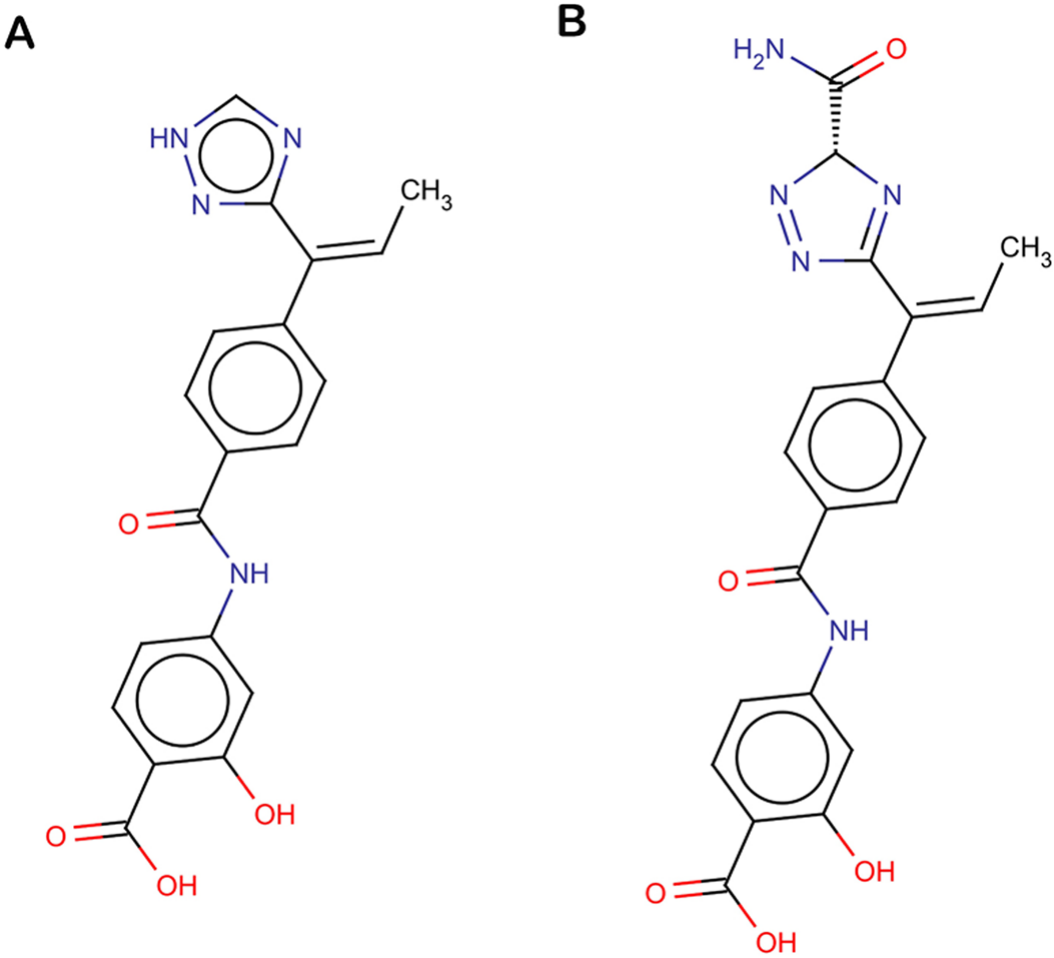
Chemical structures. (A) C_773 (B) C_997.

A set of 35 top scoring molecules, generated using growing and linking strategy were then used for molecular docking. All the molecules were first prepared using LigPrep. These molecules were then docked against the active site of COX-2 protein using the XP docking protocol of Glide. The compounds with docking score more than 8.00 (S1 Table) were then docked against the active site of COX-1 protein. Two compounds with least score for COX-1, named as C_773 and C_997 were then chosen for further consideration (S1 Table). IUPAC name of C_773 and C_997 is 5,5-dihydrogenio-3-[(1Z)-1-[4-({3-hydroxy-4-[hydroxy(λ^3^-oxidanidylidene)methyl]phenyl}carbamoyl)phenyl]prop-1-en-1-yl]-1H-1,2,4-triazol-2-ium and (3R)-3-carbamoyl-5-[(1Z)-1-{4-[(4-carboxy-3-hydroxyphenyl)carbamoyl]phenyl}prop-1-en-1-yl]-3H-1,2,4-triazol-1-ium respectively and their chemical structures is shown in Fig 4. These molecules had high binding affinity for the COX-2 binding pocket and less affinity for the active site of COX-1 enzyme. Hence, these compounds can be suggested as selective towards COX-2. C_773 had a docking score of -10.298 kcal/mol with COX-2 and -3.806 kcal/mol with COX-1. Similarly C_997 had a glide docking score of -8.688 kcal/mol with COX-2 and -3.435 kcal/mol with COX-1.

**Fig 5:**
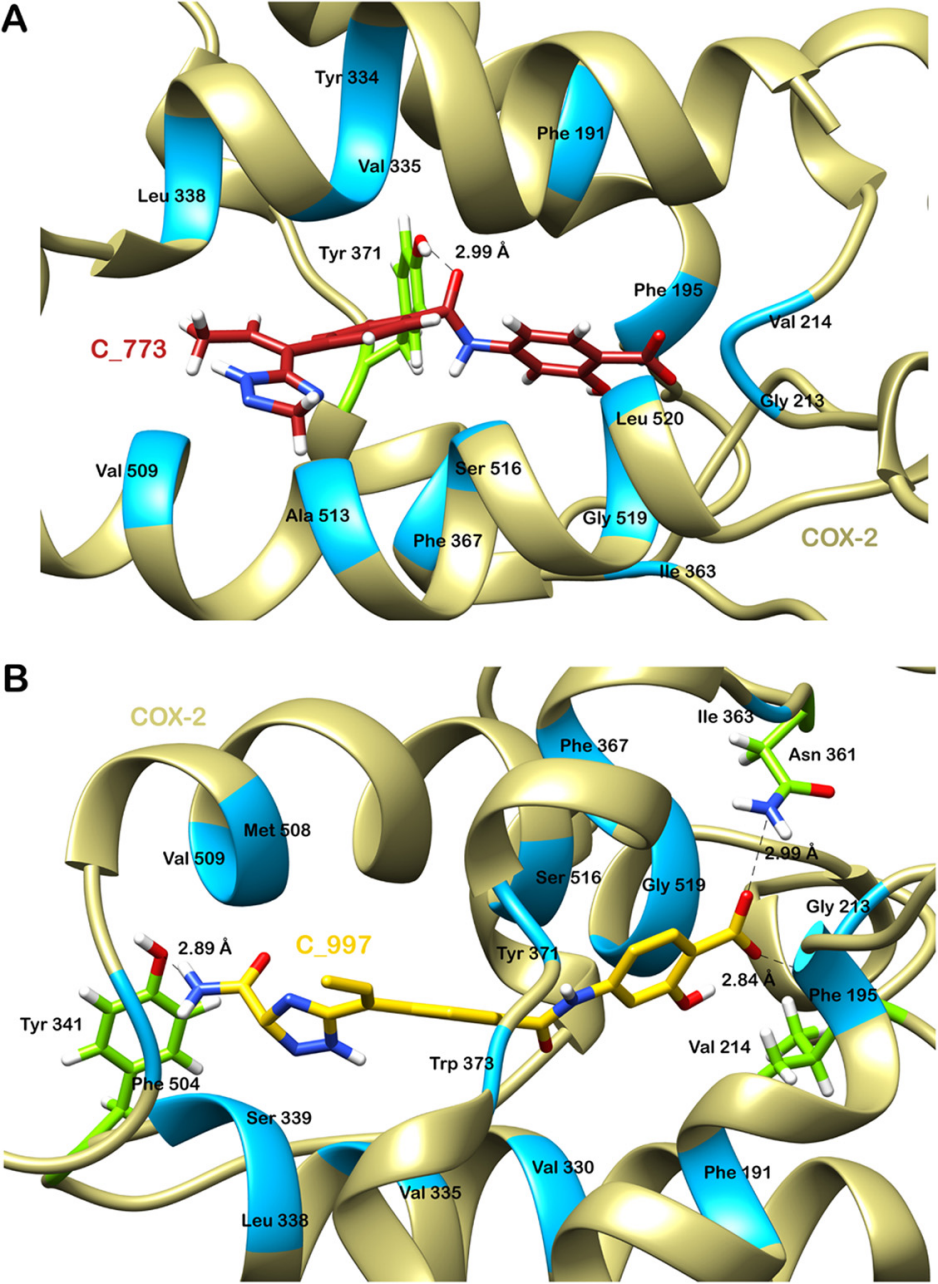
Molecular interaction pattern between ligand and protein after docking. (A) Interactions between COX-2 and C_773. (B) Interactions between COX-2 and C_997. Hydrogen bond forming residues are shown in green and hydrophobically interacting residues are shown in blue.

Ligplus program was then used to study the pattern of molecular interaction between COX-2 and both the ligands. A comparative view of scores and interacting residues between binding partners in all the complexes is given in Table 1. C_773 was forming a hydrogen bond with Tyr 371 with a bond length of 2.99 Å, which is a critical residue for COX-2 functioning [4]. Various other protein residues interacting hydrophobically with C_773 were Phe 191, Phe 195, Gly 213, Val 214, Tyr 334, Val 335, Leu 338, Ile 363, Phe 367, Val 509, Ala 513, Ser 516, Gly 519 and Leu 520 (Fig 5(A)). Most of above residues were involved in binding of COX-2 with C_997 as well (Fig 5(B)). C_997 was forming three h-bonds; one with Val 214 having a bond length of 2.84 Å and others with Asn 361 and Tyr 341 with a bond length of 2.99 Å and 2.89 Å. Many other residues lining the active pocket of COX-2 were interacting hydrophobically with C_997 to hold it within the catalytic site. These amino acids were Phe 191, Phe 195, Gly 213, Val 330, Val 335, Leu 338, Ser 339, Ile 363, Phe 367, Tyr 371, Trp 373, Phe 504, Met 508, Val 509, Ser 516, Gly 519 and Leu 520. It has been previously reported that most of the NSAIDs bind to Tyr385 and Ser530 in *Mus musculus* COX-2 which are conserved as Tyr384 and Ser529 in human COX-1 while Tyr371 and Ser516 in human COX-2), blocking the cyclooxegenase action of enzyme [49]. This clearly indicates that the two molecules identified were showing interactions with these conserved amino acids in COX-2 and hence can be foreseen to inhibit the functional activity of the target enzyme.

**Table 1:** Docking score of C_773 and C_997 with different isoforms of COX enzymes. The proteins residues involved in interaction with the ligands are also listed.

Ligand	COX-2			COX-1	
	Docking score	Protein residues interacting before MDS*	Protein residues interacting after MDS*	Docking score	Protein residues interacting* (MDS was not carried out)
C_773	-10.298	Phe 191, Phe 195, Gly 213, Val 214, Tyr 334, Val 335, Leu 338, Ile 363, Phe 367, **Tyr 371**, Val 509, Ala 513, Ser 516, Gly 519, Leu 520	Phe 195, Gly 213, Val 214, Val 330, Val 335, **Asn 361**, Ile 363, Phe 367, Leu 370, Trp 373, Met 508, Val 509, Gly 512, **Phe 515**, Ser 516, Gly 519, Leu 520, Asn 523	-3.806	Val 115, Arg 119, Val 343, Tyr 347, Val 348, Leu 351, Tyr 354, Phe 380, Tyr 384, Trp 386, Phe 512, Ile 522, Ala 526, Ser 529, Leu 530
C_997	-8.688	Phe 191, Phe 195, Gly 213, **Val 214**, Val 330, Val 335, Leu 338. Ser 339, **Tyr 341**, **Asn 361**, Ile 363, Phe 367, Tyr 371, Trp 373, Phe 504, Met 508, Val 509, Ser 516, Gly 519, Leu 520	Phe 195, His 212, Gly 213, **Asn 361**, Ile 363, Phe 367, Leu 370, Tyr 371, Phe 504, **Met 508**, Gly 512, Ala 513, Ser 516, Gly 519, Leu 520, Asn 523	-3.435	**Arg 82**, Thr 88, Leu 92. Met 112, Val 115, Arg 119, Leu 351, Tyr 354, Phe 380, Leu 383, Tyr 384, Trp 386, Phe 517, **Met 521**, Ile 522, Ala 526, Ser 529, Leu 530

* Residues in bold are the ones involved in h-bond formation.

### Investigating the dynamic stability of the docked complexes

**Fig 6:**
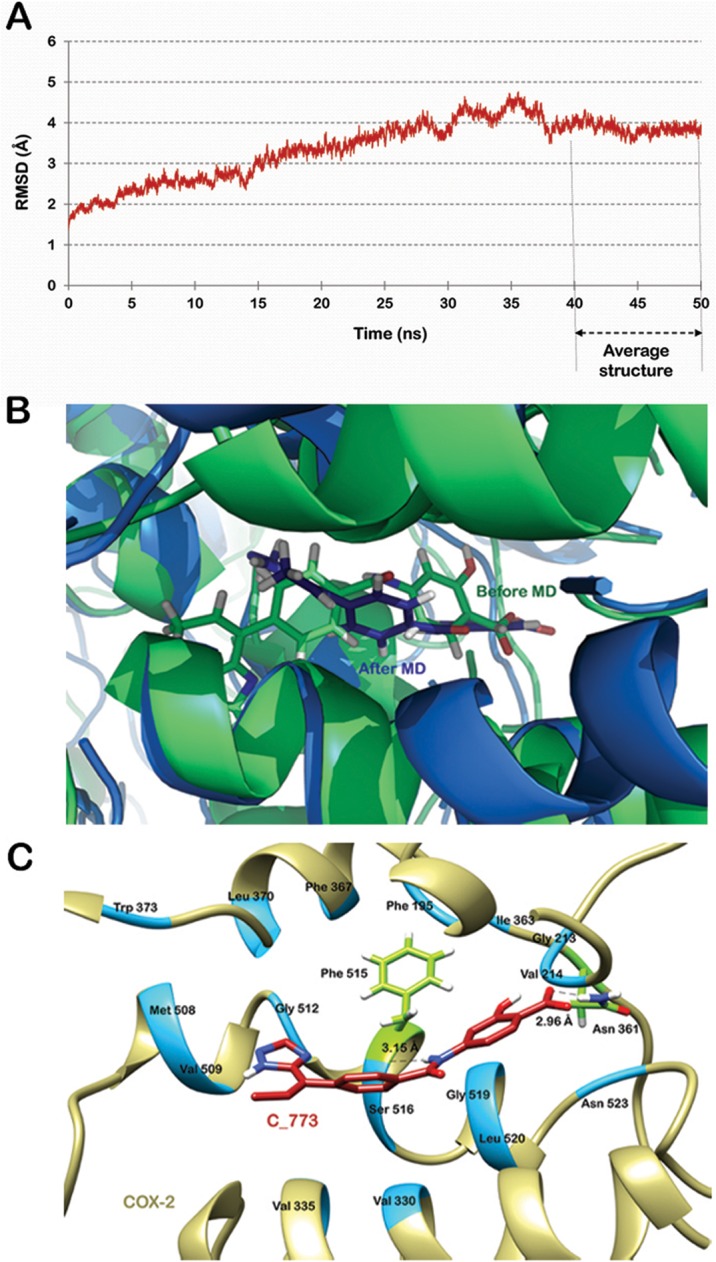
Molecular dynamics simulation of COX-2-C_773 complex. (A) RMSD trajectory of the protein backbone in reference to the structure obtained subsequent to docking. (B) Superimposition of the complex before and after simulation run. (C) Molecular interactions between the two partners. Hydrogen bond forming residues are shown in green and hydrophobically interacting residues are shown in blue.

**Fig 7:**
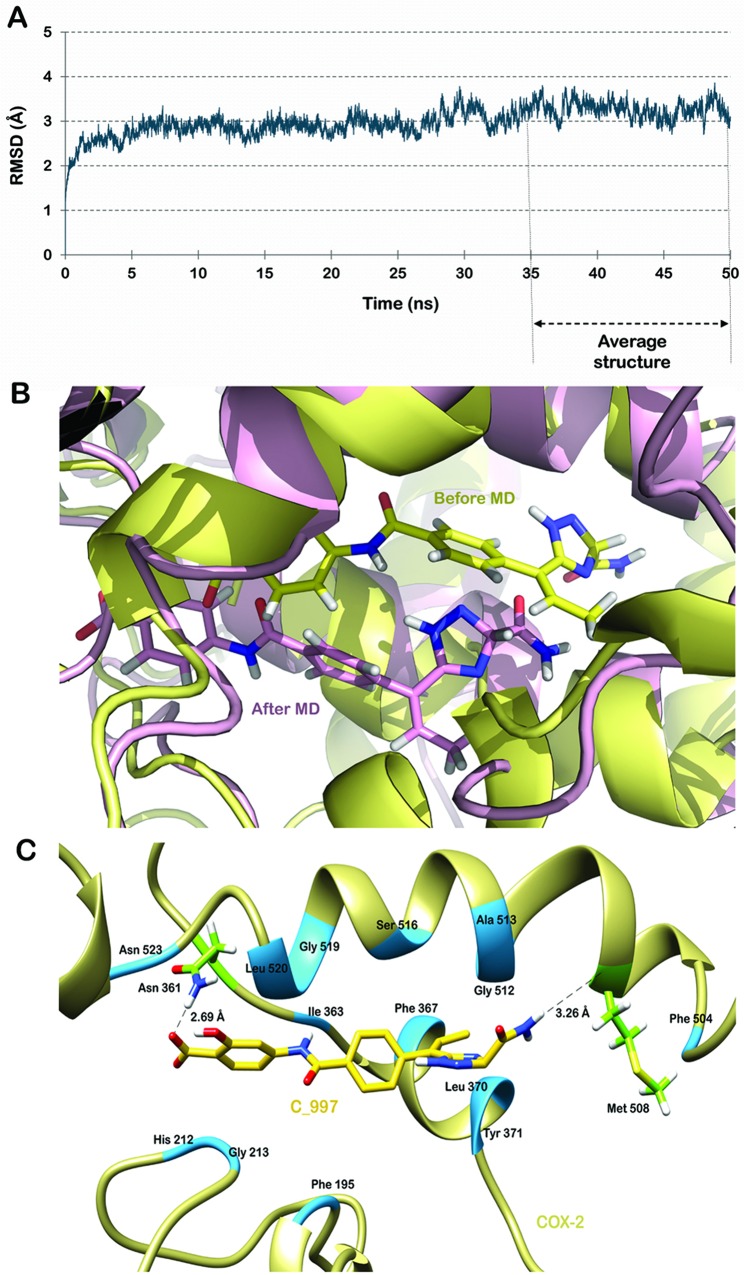
Molecular dynamics simulation of COX-2-C_997 complex. (A) RMSD trajectory of the protein backbone in reference to the structure obtained subsequent to docking. (B) Superimposition of the complex before and after simulation run. (C) Molecular interactions between the two partners. Hydrogen bond forming residues are shown in green and hydrophobically interacting residues are shown in blue.

The protein receptor is considered rigid in nature during semi-flexible docking methods. To get a more realistic picture of the interactions between protein and the ligands, the docked complexes were simulated in a water box for about 50 ns. For both the complexes, RMSD of the protein backbone in reference to the structure obtained subsequent to docking was plotted against time for the entire simulation run (Figs 6 (A) and 7 (A)). Frames were extracted from the stable trajectory between 40 to 50 ns for C_773 and 35 to 50 ns for C_997 to compute the average structure.

A slight difference was observed in the interaction pattern after the simulation run. C_773 was now forming two h-bonds with Asn 361 and Phe 515 respectively. The bond lengths were 2.69 Å and 3.15 Å (Fig 6(B)). The bond with Tyr 371 found in the docked structure weakened due to shift in the position of the docked ligand. The distance between Tyr 371 and oxygen atom of the ligand varied from 3 to 9 Å but was in between to 4 to 6 Å for most of the simulation time (S1 File). Although the ligand showed a minor movement, it was still occupying the same pocket in the protein. The other residues forming hydrophobic contacts with COX-2 were Phe 195, Gly 213, Val 214, Val 330, Val 335, Ile 363, Phe 367, Leu 370, Trp 373, Met 508, Val 509, Gly 512, Ser 516, Gly 519, Leu 520 and Asn 523. These interactions are shown in Fig 6(C). As illustrated in Fig 7(B) C_997 also changed its orientation during the MD simulation. The h-bond with Asn 361 remained conserved while the other two were lost. A new hydrogen bond emerged with Met 508 with a bond length of 3.26 Å. The other amino acids contributing in binding of C_997 with COX-2 were Phe 195, His 212, Gly 213, Ile 363, Phe 367, Leu 370, Tyr 371, Phe 504, Gly 512, Ala 513, Ser 516, Gly 519, Leu 520 and Asn 523 (Fig 7(C)).

C_773 and C_997 were occupying the site in protein which otherwise is accommodated by the natural ligands of COX enzymes. Both the ligands were also interacting with the residues participating in the reaction mechanism and therefore should block the natural process which gets stimulated upon signs leading to inflammation. Thus, we can say that these two predicted compounds possess the potential to block the binding of natural substrates of COX-2 enzyme without affecting COX-1 enzyme and hence may serve as potential inhibitor for COX-2 selectively.

### Calculation of binding free energy

The maestro pose viewer file of the two complexes: COX-2-C_773 and COX-2-C_997 was used as an input for MM-GBSA analysis. The simulated structures were used for the binding free energy estimation. The free energy of binding was estimated to be -80.063 kcal/mol for C_773 and -73.791 kcal/mol for C_997. It showed that the binding of both the ligands with the COX-2 protein will be a thermodynamically favorable process.

### Comparison with documented COX-2 inhibitors

The docking score for celecoxib was -9.878 kcal/mol, for valdecoxib was -9.458 kcal/mol and for rofecoxib was -9.367 kcal/mol. The scores were similar to docking scores of the predicted novel inhibitors, C_773 and C_997 (as shown in Table 2). Therefore we can say that the newly identified compounds can show similar inhibitory effect for the COX enzyme along with an additional advantage of being highly specific towards the COX-2 isoform of the enzyme. This selectivity with high affinity will help to overcome the limitations of the abandoned drugs.

**Table 2:** Glide docking score of the three drugs and predicted compounds when docked against COX-2.

S. No.	Drug or Ligand	Glide Docking Score
1.	Valdecoxib	-9.458
2.	Celecoxib	-9.878
3.	Rofecoxib	-9.767
4.	C_773	-10.298
5.	C_997	-8.688

### Physico-chemical properties and pharmacokinetics of C_773 and C_997

QikProp predicts significant physical descriptors and pharmaceutically relevant properties of organic molecules. It also provides significant range of values for comparing these molecular properties with those of 95% of already known pharmaceutical drugs. It gives a descriptor “#star” which denotes the number of properties of the organic molecule which do not fall within the range of values for properties of already known drugs. So, lower the number better is the druglikeness of the small molecule. The value of #star for C_773 was 0 and for C_997 was 2. Hence, only a very few computed property lied outside the required range. Lipinski’s rule of five is a thumb rule which determines the druglikeliness of a candidate molecule using four molecular properties. C_773 with a molecular mass of 366.376 g/mol, 4 hydrogen bond donors, 10.15 hydrogen bond acceptors and an octanol/water partition coefficient of 0.486 passed all the conditions of rule of five as per QikProp. C_997 had a molecular mass of 409.401g/mol. Number of hydrogen bond donors was estimated to be 6 while the count of hydrogen bond acceptors was 12.65. Predicted octanol/water partition coefficient was -1.343. With all these values falling within the recommended range of values given by QikProp, C_997 also satisfied the Lipinski’s rule. Solvent accessible surface area (SASA) and especially polar surface area (PSA) is known to have a good correlation with the passive molecular transport through membranes and therefore allows estimation of transport properties for drugs. The total SASA for C_773 was 651.808 Å^2^ with hydrophobic component of 164.237 Å^2^ and hydrophilic component of 244.494 Å^2^. SASA for C_997 was found to be 701.997 Å^2^ with hydrophobic component of 120.715 Å^2^ and hydrophillic component of 332.929 Å^2^. These values were well within the range given by QuickProp. Some of the other parameters were calculated using knowledge based set of rules through an online server, AdmetSAR. C_773 was predicted to show high human intestinal absorption with a probability of 0.78 while C_997 was predicted to show poor intestinal absorption. Blood brain barrier (BBB) is a regulatory system which separates the brain environment from the direct contact of circulatory system thereby protecting the brain for unwanted solute particles. Both the molecules were predicted to be BBB negative ensuring its administration safe for the brain. Induction of the P-glycoprotein (P-gp), an efflux transporter, leads to reduced bioavailability of orally administered drugs and can therefore decrease is therapeutic efficacy. Alternatively, its inhibition can also result in increased bioavailability, thus leading to an increased risk of adverse side effects. Thus, in general a drug should be neither a substrate nor an inhibitor of P-gp. It is expected that P-gp would not act as a barrier for the systemic exposure of C_997. On the other hand C_773 was found to be a substrate of P-gp but without inhibitory activity against it. Both the compounds were also shown to be non-carcinogenic with some levels of toxicity in Rat, fish and *Tetrahymena Pyriformis*.

### An estimate of the synthetic accessibility of the predicted compounds

‘SyntheticAccessibility’ program uses commercially available compound databases and molecular descriptors to calculate the final SA score. SA gives an estimated of the probability of existence of substructures of the query compound in the fragment database prepared from the commercially available compound databases. It also takes into consideration the number of symmetry atoms, the graph complexity, and the number of chiral centers of the compound [33]. The FA4 model was used for the calculation of SA score of all the molecules. These scores are listed in Table 3. The two predicted COX-2 selective inhibitors, C_773 and C_997 had a SA score of 4.680 and 5.036 respectively, which indicates significant synthesizability of these small molecules.

**Table 3:** SA score for all the molecules involved in the study.

S. No.	Compound	SA score
Drugs molecules used for building compound library		
1.	Diclofenac	3.203
2.	Flubiprofen	3.224
3.	Ibuprofen	2.587
4.	Indomethacin	2.585
5.	Ketoprofen	2.997
6.	Ketorolac	3.030
7.	Naproxen	2.795
8.	Piroxicam	3.374
9.	Sulindac sulfide	3.562
10.	Tenoxicam	3.293
11.	Tolmetin	3.021
FDA approved COX-2 drugs		
12.	Rofecoxib	3.475
13.	Celecoxib	3.840
14.	Valdecoxib	3.672
Predicted COX-2 selective inhibitors		
15.	C_773	4.680
16.	C_997	5.036

## Conclusions

Most of the currently available drugs are able to reduce inflammation, but cause severe side effects due to non-selective inhibition of both the isoforms of COX enzyme. Structure based computational *de novo* drug designing approach was used here to identify two highly probable selective COX-2 inhibitors to overcome the limitations posed by the drugs in clinical use. COX-2 selective compounds C_773 and C_997 satisfied drug likeliness and are predicted to have good synthetic accessibility. These attributes along with the binding mode and free energy analysis suggest that these novel compounds have the potential to serve as effective anti-inflammatory inhibitor molecules.

## Supporting Information

S1 File Distance between Tyr 371 of COX-2 and oxygen atom of C_773 during the entire simulation run. Tyr 371 is an important residue involved in the reaction which catalysis the conversion of AA to arachidonyl radical for the synthesis of prostaglandins. Although the distance between the two residues varied from 3 to 9 Å, the distance between them ranged from 4 to 6 Å for most of the simulation time as shown in the below graph.
(DOCX)

S1 Table List of molecules with docking score for COX-2 and COX-1 along with their LigBuilder scores. (DOCX)
