# Supplementary material for: Computational Structure-Based De Novo Design of Hypothetical Inhibitors against the Anti- Inflammatory Target COX-2
Source: PLoS One. 2015 Aug 4;10(8):e0134691. doi: 10.1371/journal.pone.0134691 (PMC4524694; doi:10.1371/journal.pone.0134691)
Supplement: S1 File — Tyr 371 is an important residue involved in the reaction which catalysis the conversion of AA to arachidonyl radical for the synthesis of prostaglandins. Although the distance between the two residues varied from 3 to 9 Å, the distance between them ranged from 4 to 6 Å for most of the simulation time as shown in the below graph. (DOCX) [file pone.0134691.s001.docx]

**File S1.** **Distance between Tyr 371 of COX-2 and oxygen atom of C_773 during the entire simulation run.** Tyr 371 is an important residue involved in the reaction which catalysis the conversion of AA to arachidonyl radical for the synthesis of prostaglandins. Although the distance between the two residues varied from 3 to 9 Å, the distance between them ranged from 4 to 6 Å for most of the simulation time as shown in the below graph.


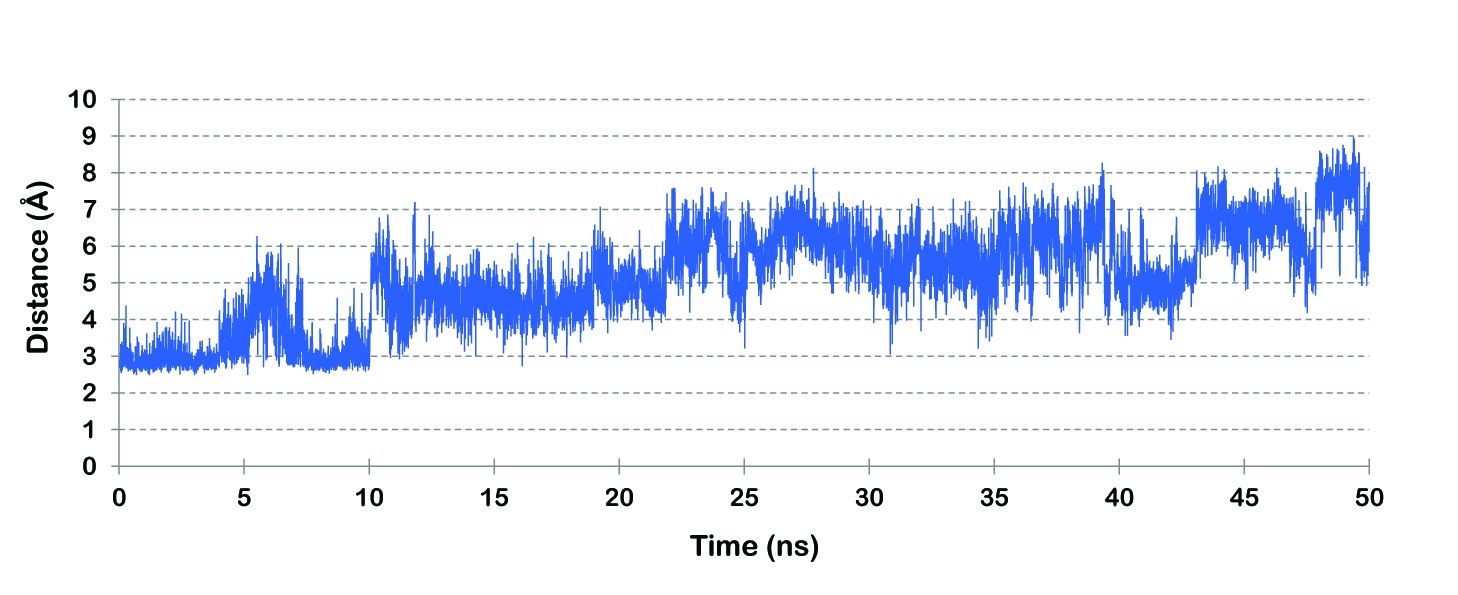


| **Time instant** | **Distance between Tyr 371 and C_773** |
| --- | --- |
| 0 ns | 2.80 Å |
| 10 ns | 5.14 Å |
| 20 ns | 5.28 Å |
| 30 ns | 6.14 Å |
| 40 ns | 4.49 Å |
| 50 ns | 6.14 Å |
