# Supplementary material for: Computational Structure-Based De Novo Design of Hypothetical Inhibitors against the Anti- Inflammatory Target COX-2
Source: PLoS One. 2015 Aug 4;10(8):e0134691. doi: 10.1371/journal.pone.0134691 (PMC4524694; doi:10.1371/journal.pone.0134691)
Supplement: S1 Table — (DOCX) [file pone.0134691.s002.docx]

**Table S1. List of molecules with docking score for COX-2 and COX-1 along with their LigBuilder scores**

| **S. No.** | **Compound** | **Docking score** | | **LigBuilder scores** | **Chemical Structure** |
| --- | --- | --- | --- | --- | --- |
|  |  | **COX-2** | **COX-1** |  |  |
| Growing Strategy | | | | | |
| 1. | C_1322 | -12.1406 | -6.33356 | 7.33 | 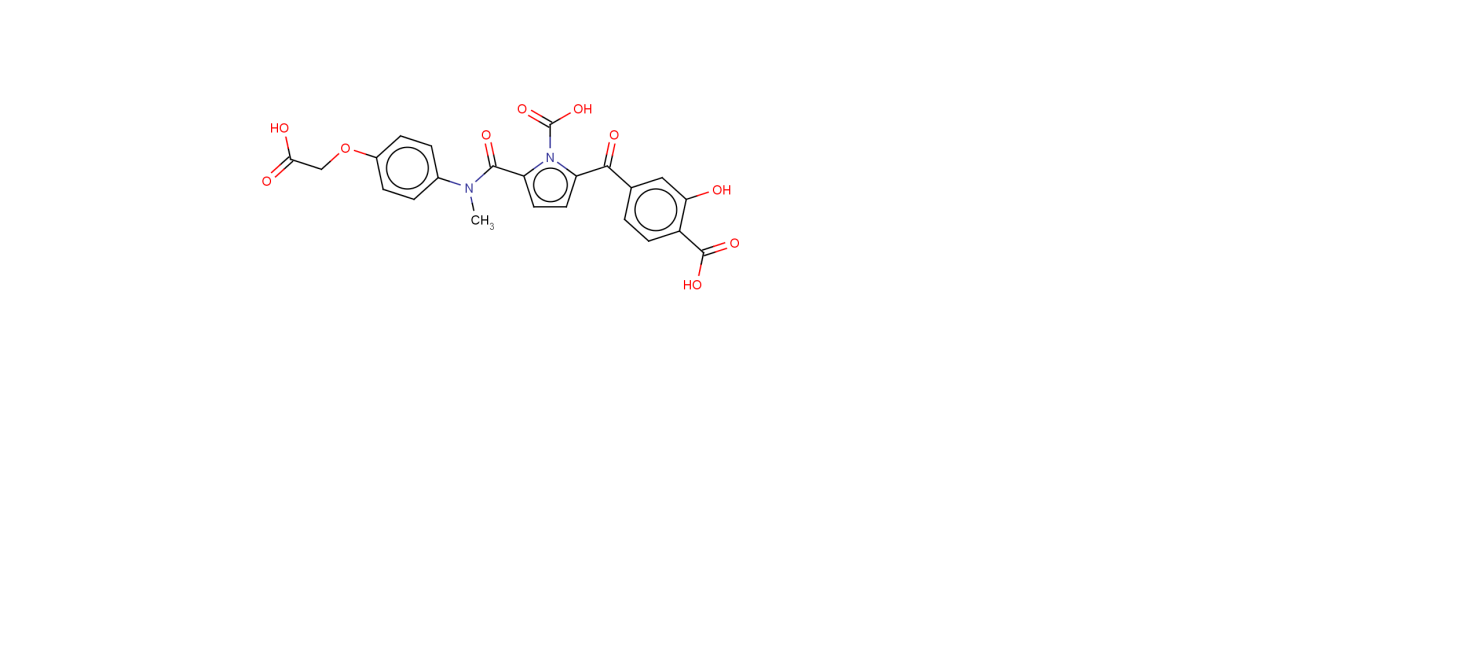 |
| 2. | C_1318 | -11.3614 | -6.03552 | 7.77 | 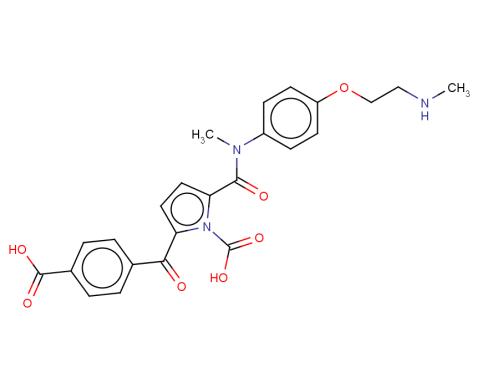 |
| 3. | C_1379 | -10.8722 | -5.49649 | 7.67 | 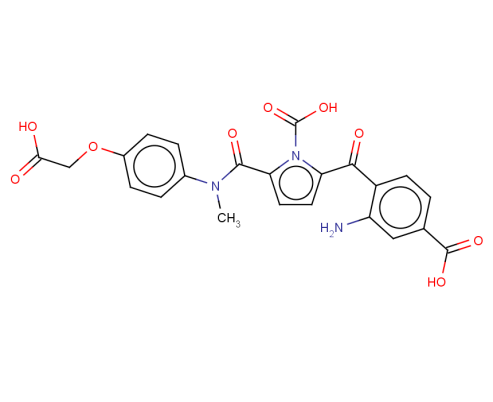 |
| 4. | C_998 | -9.30847 | -5.49995 | 7.9 | 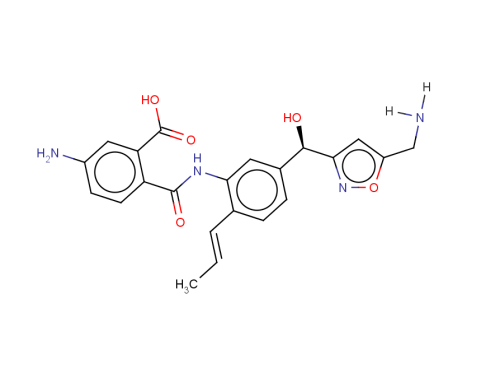 |
| 5. | C_1445 | -9.02888 | -4.70636 | 7.89 | 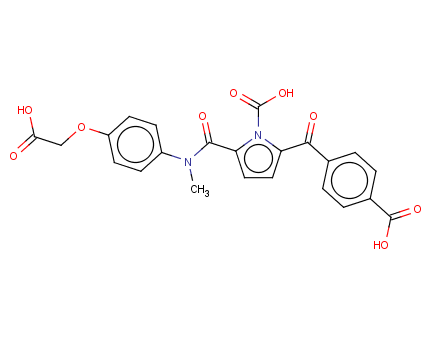 |
| 6. | C_1234 | -8.33451 | -5.38898 | 7.8 | 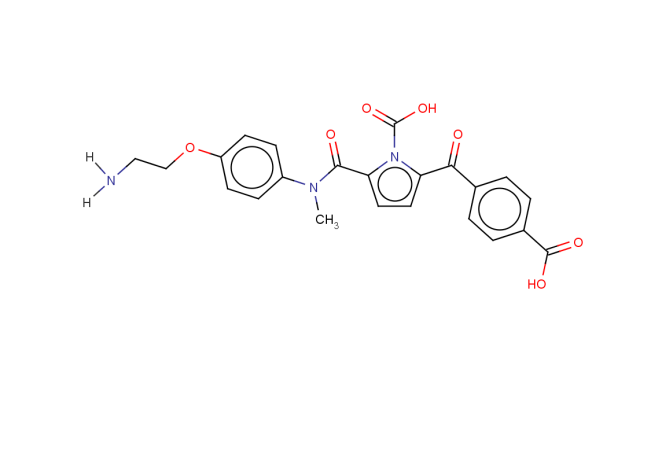 |
| 7. | C_1397 | -8.29806 | -6.40195 | 7.92 | 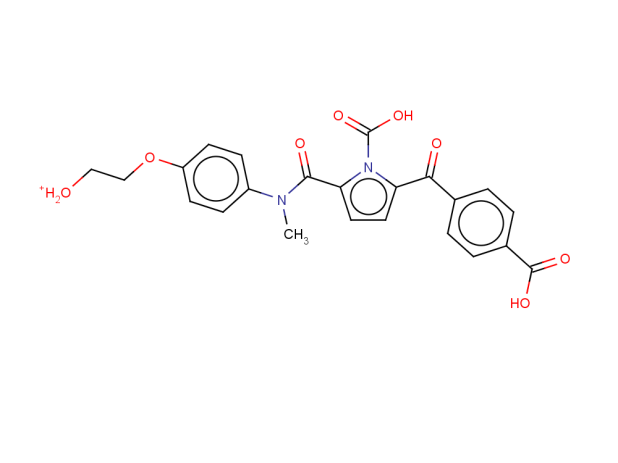 |
| 8. | C_1464 | -8.05648 | -5.56061 | 7.96 | 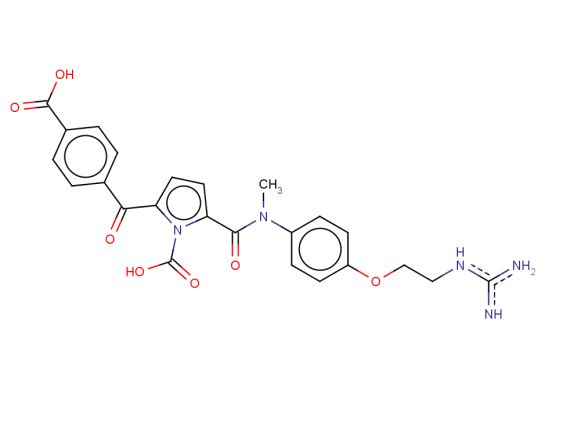 |
| Linking Strategy | | |  |  |  |
| 1. | C_976 | -12.822 | -10.076404 | 8.23 | 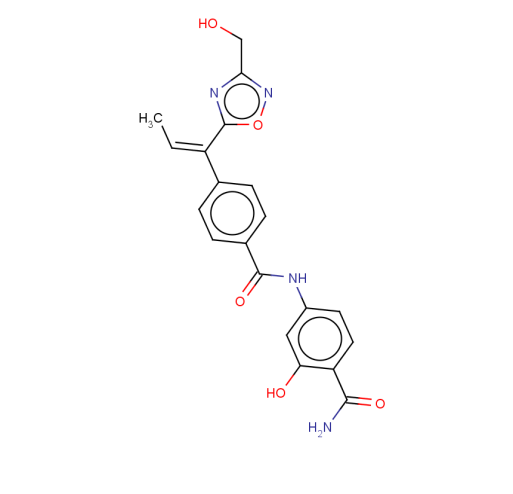 |
| 2. | C_993 | -12.7479 | -10.435137 | 8.46 | 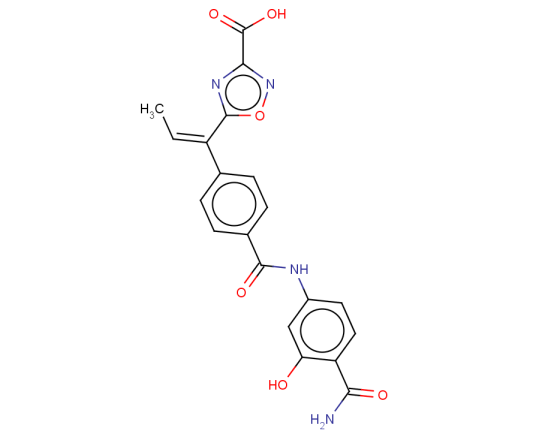 |
| 3. | C_1013 | -11.2332 | -6.66372 | 8.34 | 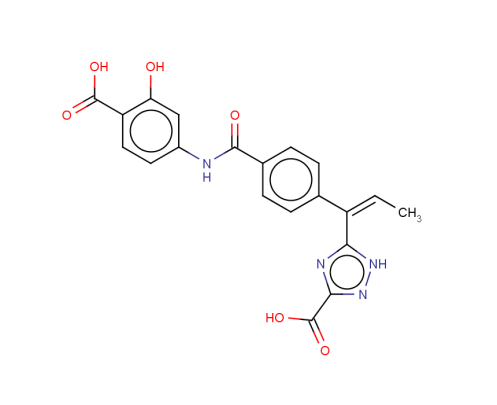 |
| 4. | C_994 | -11.0826 | -9.297963 | 8.38 | 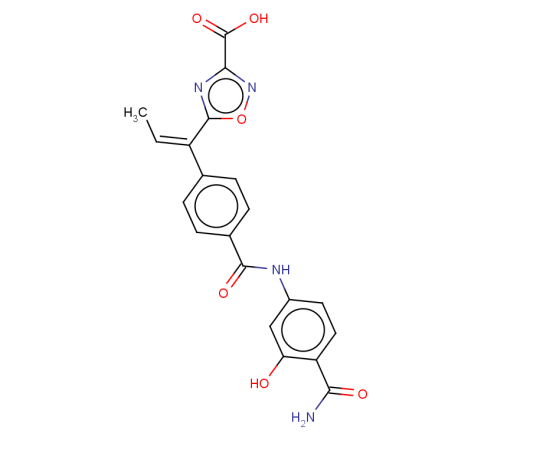 |
| 5. | **C_773** | **-10.2978** | **-3.80576** | **8.17** | **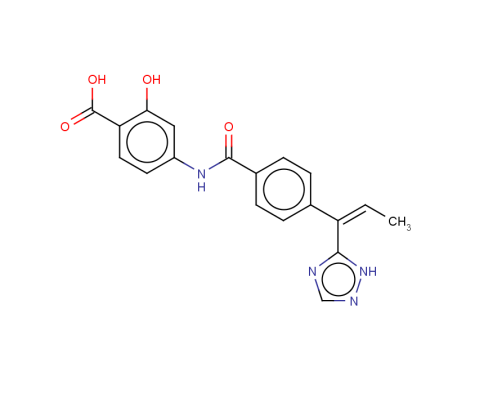** |
| 11. | C_1034 | -10.2814 | -10.946272 | 8.19 | 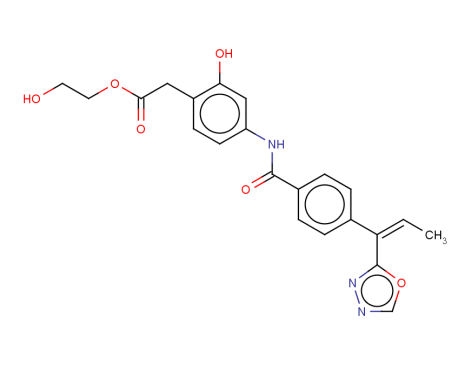 |
| 13. | C_1015 | -8.78775 | -6.73328 | 8.23 | 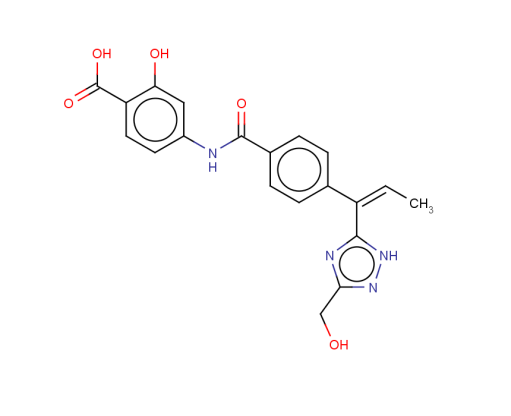 |
| 14. | C_799 | -8.7166 | -6.25835 | 7.76 | 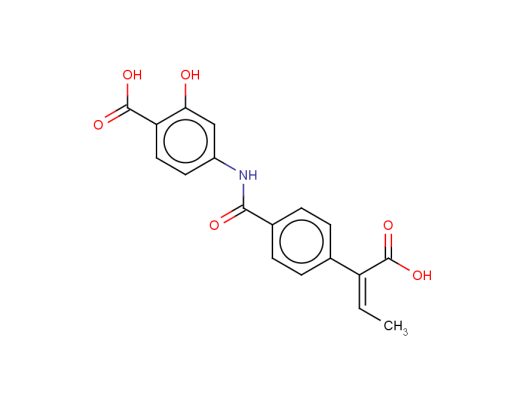 |
| 15. | **C_997** | **-8.68798** | **-3.43489** | **8.09** | **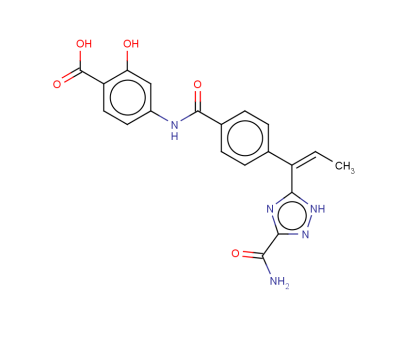** |
